# Supervised or Home-Based? Exploring the Best Exercise Approach for Knee Osteoarthritis Management: A Systematic Review and Meta-Analysis

**DOI:** 10.3390/jcm14020525

**Published:** 2025-01-15

**Authors:** Jean Mapinduzi, Gérard Ndacayisaba, Penielle Mahutchegnon Mitchaϊ, Oyéné Kossi, Bruno Bonnechère

**Affiliations:** 1 REVAL Rehabilitation Research Center, Faculty of Rehabilitation Sciences, University of Hasselt, 3590 Diepenbeek, Belgium; bruno.bonnechere@uhasselt.be; 2 TechnoRehab Lab, Filière de Kinésithérapie et Réadaptation, Département des Sciences Cliniques, Institut National de Santé Publique (INSP), Bujumbura 6807, Burundi; 3 Cabinet de Kinésithérapie et d’Appareillage Orthopédique (CKAO-AMAHORO), Bujumbura 6807, Burundi; 4 Technology-Supported and Data Driven Rehabilitation, Data Science Institute, University of Hasselt, 3590 Diepenbeek, Belgium; 5 Centre National de Référence en Kinésithérapie et Réadaptation Médicale (CNRKR), Bujumbura 6807, Burundi; ndacayisaba199065@gmail.com; 6 Ecole Nationale de Santé Publique et de Surveillance Epidémiologique (ENATSE), Université de Parakou, Parakou 03 BP 10, Benin; oyene.kossi@gmail.com (P.M.M.); mahutchegnon@gmail.com (O.K.); 7 Department of PXL-Healthcare, PXL University of Applied Sciences and Arts, 3500 Hasselt, Belgium

**Keywords:** knee osteoarthritis, supervised exercises, home-based exercises, meta-analysis, rehabilitation

## Abstract

**Background/Objective**: Knee osteoarthritis (OA) is a common and debilitating condition affecting older adults, often progressing to advanced stages and requiring total joint replacement. Exercise therapy is widely recognized as the first-line approach for the prevention and initial management of OA. This systematic review assessed the effectiveness of home-based exercises (HBEs) compared to supervised exercises in alleviating pain and reducing disability among patients with knee OA. **Methods**: A systematic search of PubMed, Cochrane Library, and ScienceDirect identified randomized controlled trials (RCTs) published between January 2001 and October 2024. Methodological quality was evaluated using the Physiotherapy Evidence Database (PEDro) scale, and a meta-analysis was conducted to quantify the efficacy of these interventions. **Results**: Ten RCTs involving 917 patients were included, ranging in moderate to high methodological quality (PEDro score: 6.3 ± 1.2). Intervention durations ranged from 4 to 12 weeks. Both supervised and HBEs were found to be effective, but supervised exercises demonstrated statistically significant improvements in pain (SMD = −0.45 [95% CI −0.79; −0.11], *p* = 0.015) and disability (SMD = −0.28 [95% CI −0.42; −0.14], *p* < 0.001) compared to HBEs. **Conclusions**: Despite the superiority of supervised exercises over HBEs, considering the cost-effectiveness and ease of implementation of HBEs, we developed recommendations to create a hybrid rehabilitation program that combines both approaches to maximize clinical outcomes.

## 1. Introduction

Osteoarthritis (OA) is one of the most prevalent and incapacitating conditions that predominantly affects older adults [1,2]. With the aging of the population, articular cartilage, which is a highly specialized tissue producing smooth, painless, and almost frictionless movement, is the most significantly affected during OA progression, with a very limited repair capacity [3]. Additionally, no strict protocol for cartilage repair and regeneration has been established to date [3]. Once the cartilage structure is compromised, osteoarthritic degeneration begins, leading to joint failure and pain as an end result [3]. It is estimated that individuals who reach the age of 85 face a 25–40% lifetime risk of symptomatic OA [1,2]. A notable 10% lifetime risk of undergoing total joint replacement due to the advanced stage of OA has been documented [1,2]. This global concern impacts over 500 million people and serves as a leading cause of limitations in adult activities, with the weight-bearing joint of the knee bearing the brunt [3,4,5,6,7].

Hallmarks of OA commonly encompass the structural and functional breakdown of both articular and abarticular components [8]. Radiographically, joint space narrowing, bony sclerosis, osteophyte formation, and articular surface deformities manifest in approximately 30% of individuals over the age of 45, with a higher prevalence in women [8]. The clinical presentation further reveals an overtime worsening pain, reduced joint mobility, altered gait patterns, lower limb proprioception and balance, muscle weakness, crepitus, intermittent effusion, inflammation, and a decline in overall health and quality of life [4,9]. This can lead to reduced physical activity, deconditioning, impaired sleep, depression, fatigue stemming from sleep disruptions, disability, diminished work productivity, and increased direct and indirect healthcare expenses [10,11].

Knee OA frequently coincides with comorbidities, potentially arising from sedentary habits, medication side effects, and the influence of inflammatory cytokines, thereby contributing to a 20% higher age-adjusted mortality rate [4,7,12].

Many risk factors, such as both joint-specific elements including aberrant joint morphology, developmental joint dysplasia, varus or valgus alignment, destabilizing muscle weakness, joint injuries, and labral tears, as well as broader factors, such as age, sex, weight, genetics, ethnicity, education level, psychological factors, occupation, and diet, have been reported in the literature [11,13]. These factors, whether joint-level or holistic, can lead to cartilage damage due to vulnerability to shear stress [13].

The literature has increasingly emphasized nonpharmacological conservative treatments as the primary approach for the initial management of OA, offering a means to mitigate disability and thereby delaying the need for surgical intervention. Within this context, active exercises have surfaced as preferred interventions [14,15].

Previous systematic reviews have provided evidence concerning the impact of manual therapy or exercise therapy on mitigating pain and disability in individuals with symptomatic knee OA [16,17,18]. However, the paucity of knee OA-specific randomized controlled trials (RCTs) investigating the comparative effects of home-based exercises (HBEs) against supervised exercises has impeded such analyses. Although two recent meta-analyses have synthesized the effects of exercise therapy irrespective of intervention and comparison modalities, the distinction between HBEs and supervised exercises requires further exploration, as their delivery modes may differ significantly [19,20]. The supervised approach presents some advantages, as it often involves generic structured group exercises under motivation, readjustments and/or encouragement from a therapist, ensuring proper techniques and injury avoidance, social interaction (group-based exercises), and access to equipment, which contrasts with the HBE mode [21]. However, supervised exercises present several limitations, such as financial, temporal, geographical, over-dependence, and anthropophobia (i.e., fear of exercising in front of/or with people) constraints [21,22]. Regarding HBEs, although some advantages, such as schedule flexibility, convenience, cost-effectiveness, and privacy, are recognized, they have some limitations in terms of supervision and motivation, limited options due to the lack of equipment, distractions from home environments, the absence of feedback, and limited progression track [21,22]. Hence, scrutinizing the comparative clinical efficacy of these interventions for this specific population is imperative. To date, no systematic review and meta-analysis has explored the comparative effects of HBEs on supervised exercises. Therefore, the main objective of this study was to evaluate the comparative effects of these interventions, specifically analyzing their impact on pain reduction and disability mitigation among individuals with knee OA.

## 2. Materials and Methods

The Preferred Reporting Items for Systematic Reviews and Meta-Analyses (PRISMA) guidelines were employed for conducting this review [23] and the protocol was registered on PROSPERO under the registration number CRD42024615303.

### 2.1. Search Strategy

A comprehensive search strategy was used, encompassing the PubMed, Cochrane Library, and ScienceDirect databases. The search targeted RCTs that were published between 1 January 2001 and 31 October 2024. The inception of this timeframe in 2001 was chosen to align with the establishment and implementation of the International Classification of Functioning, Disability, and Health (ICF) as the internationally recognized framework for characterizing health and disability [24].

The process of study identification and screening was executed by two independent reviewers (J.M. and G.N.). This involved a thorough examination of the titles, abstracts, and data associated with the studies. The screening procedure was conducted in two stages: initially, only studies with eligible full-text content were retrieved and subsequently subjected to another round of screening by the same reviewers. In addition to this systematic approach, the reference lists of the initially identified studies underwent manual scrutiny to identify any additional studies worthy of inclusion. Discrepancies were resolved through discussion or via consultation with a third reviewer (O.K.).

For reference, the principal search terms that were instrumental in this process can be found in Table S1.

### 2.2. Eligibility Criteria

The search strategy was guided by the PICO criteria, outlined as follows:

- Patients: Individuals with symptomatic knee OA confirmed by X-ray assessment.
- Interventions: HBEs encompassing hip and knee flexibility or stretching and strengthening exercises, and a range of exercises targeting knee movements, along with functional exercises.
- Comparator: The same exercises as in HBE programs, but these exercises were overseen by a qualified physiotherapist within hospital or rehabilitation center settings.
- Outcome Measures: Any outcomes assessing levels of pain and disability.
- Study Design: RCTs.

**Figure 1:**
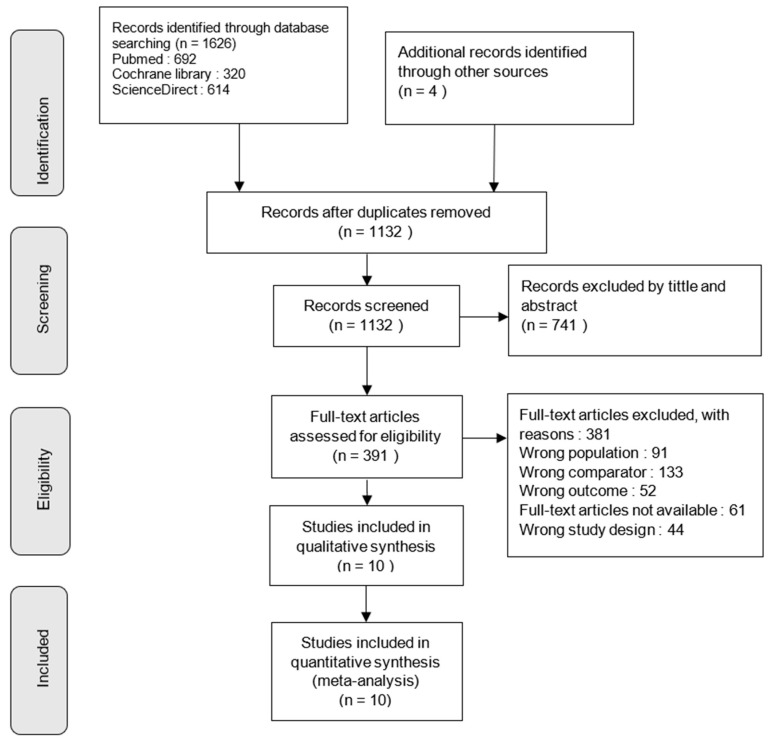
Flowchart of the study selection.

The selection process was limited to studies published in English or French. The complete flowchart of the study selection process is presented in Figure 1.

### 2.3. Quality Assessment

The PEDro scale, which is deemed a valid and reliable tool for assessing RCTs, was used for methodological quality assessment [25,26]. The quality of the RCTs was blindly judged by two different reviewers (J.M. and G.N.) to minimize potential bias [27]. In case of discordance, a third collaborator (O.K.) was consulted to provide expert input. The RCTs were classified into distinct categories based on their quality: low quality (scores falling within the range of 0 to 3 out of 10; moderate quality (scores spanning 4 to 6 out of 10); and high quality (RCTs achieving scores ranging from 7 to 10 out of 10) [27,28]. This methodical approach allowed a comprehensive evaluation of the RCTs, thus facilitating an objective assessment of their respective quality levels.

### 2.4. Data Extraction

The following information was extracted from the included studies: patient characteristics (age, gender, and BMI), number of participants, type of exercise intervention, and intervention duration, main outcome measures, and results. This information was collected, classified, and summarized by two independent reviewers (J.M. and G.N.) to guarantee the consistency of the results.

### 2.5. Data Synthesis and Analysis

Studies with comparable PICO characteristics were identified and pooled into separate meta-analyses, and their outcomes were considered for separate meta-analyses. The measure of treatment effect was the standardized mean difference effect size (standardized mean difference (SMD)), defined as the between-group difference in mean values divided by the pooled SMD computed using Hedge’s g method.

A random or fixed effect model was employed for analysis based on the heterogeneity levels among the studies, which was quantified using the I^2^ statistic [29]. Pooled estimates were computed, accompanied by their corresponding 95% confidence intervals (CIs).

The interpretation of effect sizes calculated through SMD adhered to Cohen’s classification scheme: small (0–0.20), medium (0.20–0.50), or large (0.50–0.80) [30].

Furthermore, to ascertain potential publication bias, a funnel plot was generated, and Egger’s test for the intercept was applied to assess asymmetry [31,32]. To assess the robustness of the synthesized results, we performed sensitivity analyses to identify the influence of individual studies on overall conclusions, ensuring the reliability and stability of the meta-analytic findings. Random-effect meta-regression analysis quantified the association of changes in pain and functions and the total amount of training, the frequency and the duration of the rehabilitation sessions. Studies were weighted by the inverse of the sum of the within- and between-study variance.

Statistics were performed in RStudio (version 4.3.1), and the statistical significance was predetermined at an alpha level of less than 0.05 [29].

## 3. Results

### 3.1. Patients and Study Characteristics 

Ten RCTs, representing 917 participants, were included in this analysis [33,34,35,36,37,38,39,40,41,42]. The flowchart of the study selection is presented in Figure 1.

The average age of the patients was 61.7 (6.7) years, with a mean body mass index (BMI) of 28.7 (2.2) kg/m^2^ (note that this parameter was not reported in two studies [33,36]). While six RCTs involved both genders, exhibiting a predominantly female representation (77%) [33,34,36,38,39,40], two studies exclusively considered female participants [35,37]. Notably, the sex distribution was not reported in two other studies [41,42].

The complete sociodemographic characteristics of the patients and individual studies are presented in Table 1.

The methodological quality of the included studies, as evaluated by the PEDro score, ranged from 4 to 8 out of 10, with a mean score of 6.3 (1.2). Among them, five RCTs were judged as high quality, with PEDro scores ranging from 7 to 8 [33,36,38,40,41]. The remaining studies were deemed of moderate quality, with PEDro scores ranging from 4 to 6 [34,35,37,39,42]. The complete individual scores of quality assessment are presented in Table S2.

Diverse evaluation tools and scales were employed across the included studies for patient assessment. Pain evaluation was mostly evaluated using the visual analog scale (VAS) in five studies [35,38,39,41,42], while a range of other scales were also utilized, including the numerical rating pain scale [33], the knee injury and osteoarthritis outcome score (KOOS) pain subscale [34], the brief pain inventory (BPI) [40], and Western Ontario and McMaster Universities Osteoarthritis Index (WOMAC) pain subscale [36].

Physical function evaluation involved the use of WOMAC in seven studies [33,35,36,37,40,41,42], the 6 min walk test (6MWT) in two studies [38,39], and the KOOS in another study [34]. A thorough overview of these tools is presented in Table 1.

### 3.2. Description of the Exercise Interventions

All the included studies implemented specific HBE programs compared to supervised exercises. Comprehensive details regarding the interventions’ specifics are presented in Table 1, encompassing both the content and structure of these exercise programs.

Briefly, a broad range of exercises were used, each tailored to target specific muscle groups, including those surrounding the knee, hip, and ankle. The exercise repertoire included proprioceptive training exercises, static cycling, stretching routines, walking regimens, and exercises involving both open and closed-kinetic chains (squats and calf raise, as tolerated without pain). Notably, emphasis was also placed on stabilization and neuromuscular control exercises, with focal points on the knee and hip regions.

For HBE, these exercises were independently performed within a living environment, while in the supervised group, a qualified physiotherapist controlled the sessions, ensuring optimal execution and adherence.

The overall number of sessions across all the included studies ranged from 12 to 48, with a median of 18 [16,17,18,19,20,21,22,23,24] sessions per study. The duration of the exercise programs exhibited variability, spanning from 4 to 12 weeks, with a mean duration of 7.1 (2.6) weeks. The frequency of exercise sessions ranged from 1 to 6 times per week, with a mean of 3.3 (1) times per week. Each exercise session lasted from 30 to 60 min per day, with a median of 45 [30,31,32,33,34,35,36,37,38,39,40,41,42,43,44,45,46,47,48,49,50,51,52,53,54,55,56,57,58,59,60] min per session.

### 3.3. Clinical Efficacy

To quantify the impact on pain and disability levels, a meta-analysis was conducted to compare the efficacy of HBEs and supervised exercises.

As a preliminary step, potential publication bias was evaluated. The analysis involved an analysis of the funnel plot, which did not reveal significant asymmetry (Figure S1). Furthermore, the statistical assessment using Egger’s intercept yielded a value of −0.42 (*p*-value = 0.06) for pain and 0.29 (*p*-value = 0.78) for disability. In the sensitivity analysis, no evidence of result bias was identified, indicating the robustness of our findings (Figures S2 and S3).

**Figure 2:**
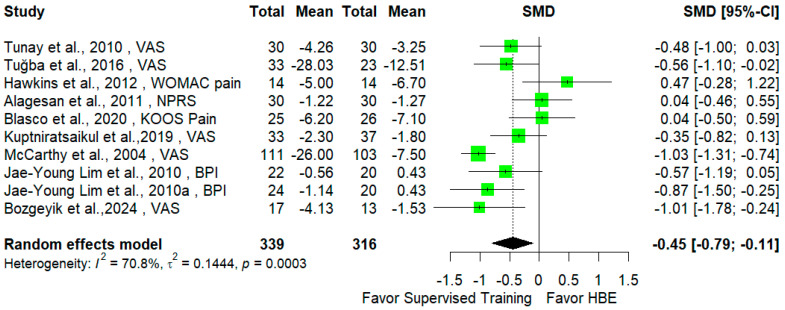
Effects of supervised exercises versus HBEs on pain.

**Figure 3:**
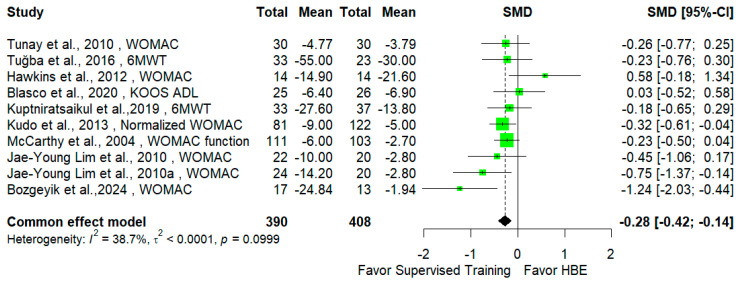
Effects of supervised exercises versus HBEs on disability.

The analysis revealed a significant difference between these two interventions with respect to pain improvement (SMD = −0.45 [95% CI −0.79; −0.11], *p* = 0.015) and disability mitigation (SMD = −0.28 [95% CI −0.42; −0.14], *p* < 0.001), both in favor of the supervised exercise intervention. Forest plots are presented in Figure 2 and Figure 3.

**Figure 4:**
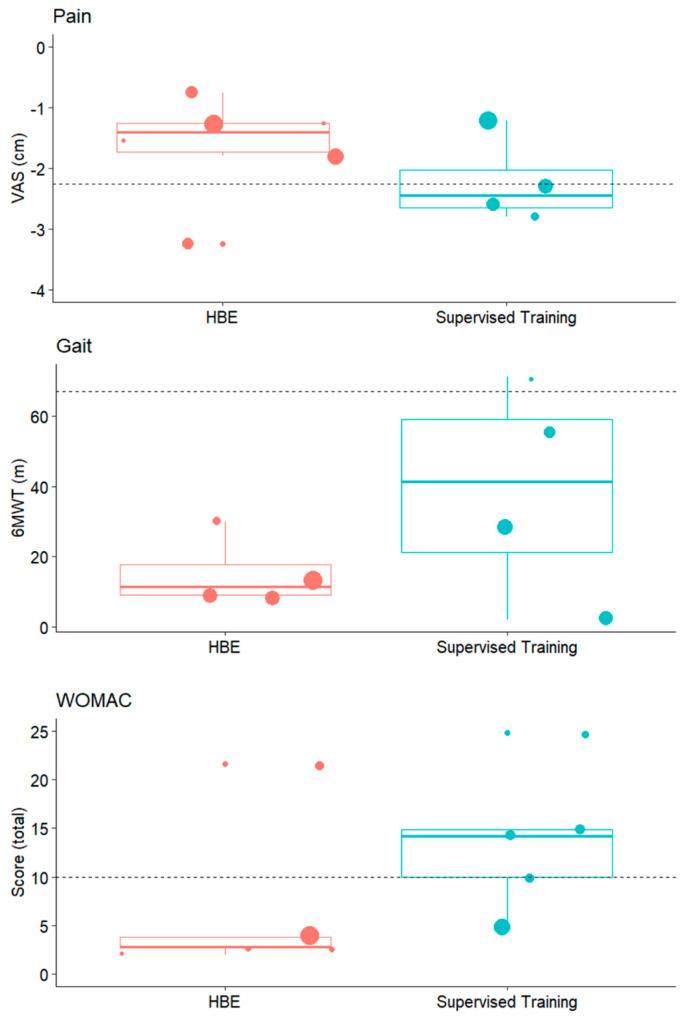
Comparative clinical improvements of HBEs and supervised exercises on pain and disability levels.

To further evaluate the clinical efficacy of HBE programs compared to supervised exercises, we examined the primary effects of each intervention and compared them to the minimal clinically important difference (MCID) for key outcomes. Pain was assessed using the VAS [35,38,39,41,42], gait performance was evaluated with the 6MWT [38,39], and functional outcomes were measured using the WOMAC [33,35,36,37,40,41,42]. By comparing the observed improvements with these established thresholds for clinical relevance, we aimed to determine whether the interventions produced meaningful benefits for participants. The findings, presented in Figure 4, show that for the VAS and the WOMAC, only the supervised exercises led to a larger improvement than MCID. On the other hand, concerning the 6MWT, neither of the two interventions led to clinically relevant differences.

### 3.4. Dose–Response Relationship

**Figure 5:**
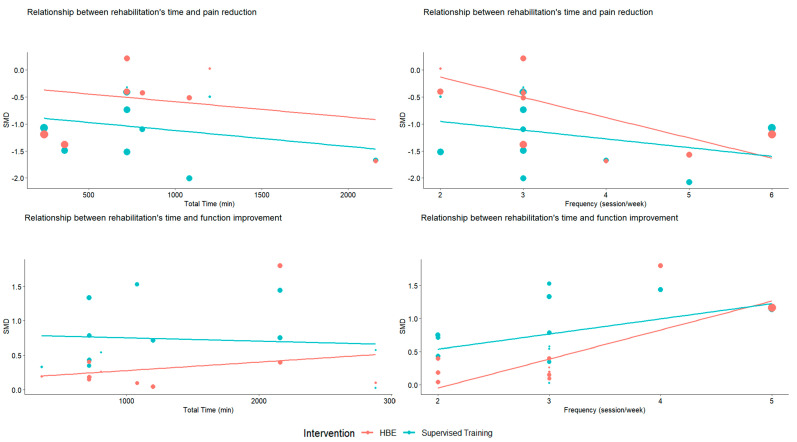
Dose–response relationship between HBEs and supervised exercises on pain and disability, for pain negative results indicate pain reduction, while for function, positive results are associated with improvement.

Next, we investigated a potential dose–response relationship by analyzing the effects of session duration, intervention frequency, and total intervention duration on outcomes. The results, summarized in Table 2 and Figure 5, reveal significant associations between clinical outcomes and the frequency of the session for both pain (β = −0.268, SE = 0.119, *p* = 0.03) and function (β = 0.334, SE = 0.113, *p* = 0.007). Interestingly, when comparing HBE and supervised exercises, these relationships were only significant for the HBE group. Concerning function, we observed a significant and positive association between total duration and functional improvement (β = 0.001, SE = 0.001, *p* = 0.04). These findings highlight the importance of intervention frequency and cumulative training volume in optimizing outcomes, particularly for HBE programs.

## 4. Discussion

This review aimed to investigate the comparative effects of HBEs and supervised exercises on pain and disability in patients with knee OA. Our meta-analysis of 10 RCTs involving 917 participants with radiographically confirmed symptomatic knee OA demonstrated that supervised exercises were more effective than HBEs in reducing pain and disability. These findings are consistent with recent studies that showed that supervised exercises were superior to HBEs, as both are effective in the management of knee OA [43,44]. In contrast, a previous study reported similar effects of supervised exercises and HBEs in reducing pain and disability severity among individuals with knee OA [45].

However, the effect sizes for these differences were small, suggesting that while supervised exercises provide an edge, HBEs remain a valuable option.

The observed superiority of supervised exercises can be explained by their structured nature, which includes professional guidance to ensure correct technique, real-time feedback, and progressive adjustments tailored to the patient’s functional status [21,22]. These factors enhance motivation and adherence, leading to better outcomes. In contrast, HBEs often lack supervision, which may result in inconsistent performance, reduced adherence, and limited progression, thereby diminishing their efficacy [21,22]. Interestingly, the analysis identified a positive association between total intervention duration and functional improvement in the HBE group, emphasizing the potential of HBEs if adherence and exercise volume are optimized. However, adherence data for HBE programs were inconsistently reported, with only three studies (30%) providing relevant information [38,40,41]. Inconsistent conclusions about this parameter were reported across the three studies. In fact, poor adherence to HBEs was reported in two studies (20%), with patients believing that potential pain flare and discomfort while exercising were the main reasons for low adherence to exercises [40,41]. In contrast, the other study (10%) found no significant difference between supervised and HBE intervention groups relative to exercise compliance, and this may help emphasize the importance of exercise therapy among patients with knee OA [38]. However, dropout rates associated with having to attend supervised sessions were reported in this study, with a relatively low number (12.5%). Since adherence is a critical determinant of exercise effectiveness, future research should prioritize strategies to monitor and enhance adherence in HBE programs. Indeed, HBE intervention is seen as a suitable option, especially in the context of limited resources, such as low-and middle-income countries (LMICs). In this context, HBE programs play a pivotal role and help overcome contextual factors unique to these regions, including limited access to healthcare facilities (lack of access to fitness centers or rehabilitation facilities, elimination of the need for travel or specialized equipment, lack of qualified healthcare professionals, scarce of public transports, etc.), time and financial constraints (elimination of transportation-related fees and time, low cost or freeliness of HBEs), and cultural considerations, where public exercise may be stigmatized or seen as a taboo [46,47].

The limitations of this review must be acknowledged and discussed before further delving into the implications for rehabilitation. First and foremost, the majority (50%) of included studies evaluating the effects of HBEs exhibited moderate-quality PEDro scores ranging from 4 to 6 out of 10 [34,35,37,39,42]. This could have introduced bias, potentially impacting the review’s level of evidence. Furthermore, the vast majority of the included studies (92%) lacked blinding of participants and therapists, a well-known limitation in rehabilitation studies. In terms of study characteristics, HBEs exhibited slight design variations. These exercises involved strength training for muscles around the knee, hip, and ankle; proprioceptive training; static cycling; stretching; open and closed-kinetic chain exercises; and stabilization or neuromuscular control exercises. These interventions targeted the knee and pelvic girdle muscles for varying durations, ranging from 4 to 12 weeks. This underscores the need for further studies exploring diverse intervention types, including exercise dosage in terms of duration, intensity, frequency, and volume, as analyses displayed a dose–response relationship in exercise programs where intervention dosage was associated with improvements, especially in HBE groups. This dosage aspect has been evidenced by recent studies as a crucial element in the rehabilitation field [48,49]. In fact, dose–response relationship is essential in the fields of rehabilitation sciences and offers the mean to explore how various doses of exercise in terms of frequency, intensity, duration, volume, and type influence health outcomes and rehabilitation benefits [50,51]. It is therefore important for clinicians to determine an individualized optimal exercise dosage based on individual characteristics, such as age, sex, fitness level, health status, specific health conditions, and response to previous exercise therapies [51]. However, determining optimal exercise dose may be a challenge due to variability in individual response to exercises, difficulty standardizing intensity across patients with different clinical presentations, and long-term adherence to prescribed exercise programs [51]. By understanding the principles of therapeutic exercises and dose–response, therapists should design effective and personalized interventions to maximize rehabilitation outcomes while minimizing risks. Furthermore, future studies should perform long-term follow-up analysis to investigate the long-term sustainability of rehabilitation outcomes and develop individualized and standardized intervention protocols for diverse patient groups, taking into account differences in health systems [12,52]. This will facilitate the contextually adapted implementation of results in countries with different levels of resources alongside more precise comparisons between studies. Additionally, most of the included studies did not report on exercise adherence; only three studies reported on exercise adherence based on the number of completed sessions and the duration of each session [38,40,41]. Importantly, evidence clearly indicates that the effectiveness of patient exercises depends on adherence, with approximately 70% of patients with chronic musculoskeletal conditions failing to consistently engage in prescribed exercise regimens [53,54]. Given the meta-analysis’s limitations, stemming from the small sample size (only 10 studies per analysis), the analyses may be subject to potential low power to generalize the findings, warranting cautious interpretation of the results. Finally, the search strategy considered only studies published in English or French language and was limited to three databases.

Despite these limitations, we found statistically and medically relevant effects of supervised exercises but also positive effects—although less important—of HBE. Therefore, a hybrid model combining supervised and HBEs could offer a promising solution to maximize benefits [55]. Such an approach can leverage the strengths of both modalities: supervised sessions provide individualized feedback and ensure proper technique, while HBEs allow schedule flexibility, convenience, and cost-effectiveness [56,57]. This combination can address the geographical, financial, and temporal limitations of supervised exercises, thereby optimizing rehabilitation outcomes.

To enhance the effectiveness of HBE programs, clinicians should focus on improving patient motivation and engagement. Strategies such as patient education, regular follow-ups through phone calls or counseling, and the integration of innovative tools like mobile apps or serious games could significantly improve adherence [58,59]. These tools can provide reminders, real-time feedback, and engaging exercise formats, making rehabilitation more accessible and enjoyable [60]. For patients with low adherence or severe pain-related avoidance behaviors, standalone supervised exercises may still be the best option.

To not overgeneralize the results of this meta-analysis, it is important to note that nearly all the included studies were conducted in high-income countries, except for one study carried out in a middle-income country, namely India [33]. Yet, detailed information about participants’ demographic characteristics, such as ethnic diversity and physical activity levels, was lacking in the included studies. Therefore, the generalizability of these findings to countries with different healthcare systems, care organizations, health literacy levels, beliefs, and cultural backgrounds requires cautious consideration. This underscores the necessity of conducting research and implementing findings in different resource settings while accounting for not only contextual realities but also demographic characteristics [61]. Notably, exercise therapies, owing to their straightforward applicability and lack of special material requirements, could be readily adapted and integrated into such settings. Additionally, the introduction of community-based rehabilitation programs in countries with limited rehabilitation access can aid the growing number of individuals with OA [62]. Community-based rehabilitation offers an affordable and accessible means of addressing complex social, economic, and clinical needs in resource-poor settings, where family members play a pivotal role in patients’ illness experience, self-efficacy, and recovery [63].

The findings of this review emphasize the need for further research to address critical gaps, particularly in understanding adherence strategies for HBEs and exploring their application in LMICs. Community-based rehabilitation (CBR) programs and the integration of innovative tools like mobile applications and serious games could further enhance the accessibility and effectiveness of HBE programs [59]. However, the combination of CBR and innovative tools may present several limitations in the context of LMICs, such as the lack of technology accessibility due to the related cost, and internet connectivity and power supply issues, especially in remote areas [64,65]. Further, the lack of user literacy and adequate training for CBR field workers on using these innovative tools, alongside cultural and language barriers, should be acknowledged as potential limitations to the adoption of this approach [64,65]. Some simplification ways, such as content standardization using one-size-fits-all approaches, usage of basic metrics like step count or symptom checklists, and predefined rehabilitation interventions that limit customization for specific patient needs, have been documented through the literature [65]. Future research directions should take into account equitable access to innovative tools, internet connectivity, and power supply through partnerships with different levels of policymakers, along with the creation of subsidized or low-cost programs for marginalized populations. Additionally, piloting scalable contextually adapted programs that can also adapt to different health conditions.

Finally, while both interventions are effective, tailoring exercise programs to individual needs and contexts will be crucial for optimizing outcomes and improving the quality of life for patients with knee OA. These results highlight the importance of individualized, flexible rehabilitation strategies and pave the way for more inclusive and accessible exercise therapies across diverse healthcare settings.

## 5. Conclusions

This systematic review and meta-analysis provide valuable insights into the comparative effects of HBEs and supervised exercises for managing pain and disability in patients with knee OA. While supervised exercises demonstrated greater efficacy, particularly in reducing pain and disability in knee OA, the differences were modest, highlighting the potential role of HBEs as a practical alternative when supervised sessions are not feasible. Notably, the positive association between intervention dose and functional improvements in the HBE group underscores the importance of adherence and exercise dosage in optimizing clinical outcomes.

A hybrid approach that combines supervised and HBE seems to emerge as a promising solution to maximize rehabilitation benefits. Supervised sessions offer personalized feedback, ensure proper technique, and provide motivation, while HBEs deliver schedule flexibility, convenience, and cost-effectiveness. This model could overcome the geographical, financial, and temporal barriers of standalone supervised exercises and enhance patient adherence and engagement.

Future studies are needed to investigate the sustainability of rehabilitation benefits at long-term follow-up and develop standardized intervention protocols for diverse patient groups and contexts.

## Figures and Tables

**Table 1:** Characteristics of the individual included studies.

Study	Country	PEDro Score	Purpose	Patients		Duration and FU Period	Outcome Measures	Main Results
				HBE	Supervised			
Tunay et al., 2010 [42]	Turkey	5/10	To establish the effects of hospital and HBE on proprioception, pain, and function in patients with knee OA	n = 30 (sex NS) Age: 54.4 ± 8 BMI: 28.8 ± 5.1	n = 30 (sex NS) Age: 50.2 ± 9.1 BMI: 27.5 ± 4.3	6 weeks 5 times/week	WOMAC VAS MFSS TUG	Both hospital and HBE decreased joint symptoms and improved function in patients with knee OA.
Tuğba et al., 2016 [39]	Turkey	6/10	To compare the effects of low-intensity exercises for lower limbs, either supervised or at home, on pain, function, and the hemodynamic parameters of knee OA patients	n = 23 (15 F) Age: 59 (51–80) BMI: 30 ± 4	n = 33 (24 F) Age: 60 (49–84) BMI: 32 ± 6	6 weeks 3 times/week	VAS (mm) 6-MWT (m) Balance score	Group-supervised exercises were more effective than HBE in reducing pain levels and improving quadriceps and hamstring muscle strength, both being effective in improving rest pain and 6 MW distance in knee OA.
Kudo et al., 2013 [37]	Japan	4/10	To evaluate the effects of the mode of treatment delivery on the improvement of symptoms in knee OA and to analyze potential risk factors affecting improvement after exercise therapies	n = 128 (F) Age: 66 ± 6 BMI: 23.8 ± 3.0	n = 81 (F) Age: 64 ± 6 BMI: 23.8 ± 2.9	12 weeks twice/week	Normalized WOMAC	A significant improvement in WOMAC was observed in supervised exercises compared with HBE.
Hawkins et al., 2012 [36]	England	7/10	To investigate the effects of supervised exercises in reducing pain and improving function compared to HBE in knee OA	n = 15 (9 F) Age: 58 ± 11 BMI: NS	n = 17 (9 F) Age: 63 ± 7 BMI: NS	12 weeks 4 times/week	WOMAC	Both groups had reduced pain and disability at week 12, with the supervised group demonstrating better outcomes than HEP.
Alagesan et al., 2011 [33]	India	7/10	To determine the effectiveness of supervised exercises versus HBE for knee OA	n = 30 (17 F) Age: 49 ± 3 BMI: NS	n = 30 (18) Age: 50 ± 3 BMI: NS	8 weeks 6 times/week	NPRS WOMAC	A significant decrease in pain and disability in favor of supervised exercises was found, with both interventions being effective.
Blasco et al., 2020 [34]	Spain	6/10	To assess the effects of preoperative balance training on the early postoperative balance and functional outcomes after total knee replacement	n = 26 (19 F) Age: 72.3 ± 4.5 BMI: 30.8 ± 5.7	n = 25 (19 F) Age: 70.2 ± 7.2 BMI: 32.5 ± 4.9	4, 6 weeks 3 times/week	KOOS	Preoperative balance training, conducted either as a domiciliary or as an outpatient, is an effective approach to enhance early postoperative balance outcomes.
Kuptniratsaikul et al., 2019 [38]	Thailand	8/10	To investigate the efficacy of underwater treadmill exercises on pain and functional improvement in obese patients with knee OA	n = 40 (37 F) Age: 61.7 ± 6.9 BMI: 28.4 ± 3.0	n = 40 (38 F) Age: 62.1 ± 6.4 BMI: 28.9 ± 3.2	4 weeks 3 times/week	VAS 6MWT	Exercise using an underwater treadmill was found to be as efficacious as HBE for relieving pain and improving function in obese people with knee OA.
McCarthy et al., 2004 [41]	UK	7/10	To compare the effectiveness of providing a HBE program versus an 8-week CBE program in patients with knee OA	n = 111 (sex NS) Age: 64.5 ± 9.9 BMI: 29.4 ± 5.2	n = 103 (sex NS) Age: 64.9 ± 9.7 BMI: 30.2 ± 5.3	8 weeks twice/week	VAS WOMAC	The supplementation of a HBE program with a CBE program led to clinically significant superior improvement. These improvements were still evident at the 12-month review.
Jae-Young Lim et al., 2010 [40]	Korea	7/10	To investigate the effectiveness of AQE and LBE on body fat, functional fitness, and functional status	n = 24 (21 F) Age: 63.3 ± 5.3 BMI: 27.7 ± 2.0	n = 25 (21 F) Age: 67.7 ± 7.7 BMI: 27.6 ± 1.7	8 weeks 3 times/week	BPI WOMAC	LBE is more effective than HBE in reducing pain and improving disability.
Jae-Young Lim et al., 2010_a_ [40]	Korea	7/10	To investigate the effectiveness of AQE and LBE on body fat, functional fitness, and functional status	n = 24 (21 F) Age: 63.3 ± 5.3 BMI: 27.7 ± 2.0	n = 26 (23 F) Age: 65.7 ± 8.9 BMI: 27.9 ± 1.5	8 weeks 3 times/week	BPI WOMAC	AQE is more effective than HBE in reducing pain and improving disability.
Bozgeyik et al., 2024 [35]	Turkey	6/10	To compare the effectiveness of supervised and home-based exercises in knee OA	n = 13 (F) Age: 56.23 ± 7.88 BMI: 28.87 ± 7.54	n = 17 (F) Age: 59.1 ± 6.7 BMI:28.7 ± 5.2	6 weeks 3 times/week	VAS WOMAC	The physiotherapist-supervised exercises had better effects on pain and knee function than the home-based exercises.

AQE = aquatic exercise; BMI = body mass index; BPI = Brief Pain Inventory; CBEs = class-based exercises; F = female; FU = follow-up; HBE = home-based exercises; KOOS = Knee injury and Osteoarthritis Outcome Score; LBE = land-based exercise; MFSS = Monitorized Functional Squat System Proprioceptive Test; 6MWT = 6-Minute Walk Test; n = number; NPRS = Numerical Pain Rating Scale; NS = not specified; OA = osteoarthritis; TUG = Timed Up and Go test; VAS = Visual Analogue Scale; WOMAC = Western Ontario and McMaster Universities Osteoarthritis Index.

**Table 2:** Dose–response relationship between HBEs and supervised exercises on pain and disability.

Outcome	Condition	Duration (One Session)		Frequency		Duration (Total)	
		β (SE)	*p*	β (SE)	*p*	β (SE)	*p*
PAIN	Supervised Training	−0.001 (0.011)	0.93	−0.162 (0.165)	0.35	−0.0003 (0.0003)	0.45
	HBE	0.009 (0.13)	0.47	**−0.375 (0.148)**	**0.03**	−0.0003 (0.0004)	0.22
	TOTAL	0.004 (0.009)	0.63	**−0.268 (0.119)**	**0.03**	−0.0002 (0.0002)	0.33
FUNCTION	Supervised Training	0.003 (0.008)	0.081	0.229 (0.158)	0.17	−0.00005 (0.0001)	0.77
	HBE	0.0007 (0.008)	0.93	**0.538 (0.136)**	**0.008**	**0.001 (0.001)**	**0.04**
	TOTAL	0.002 (0.006)	0.77	**0.334 (0.113)**	**0.007**	0.00004 (0.0001)	0.76

For pain, negative results indicate pain reduction, while for function, positive results are associated with improvement.

## Data Availability

No new data were generated during this study (systematic review). However, the aggregated data are available upon reasonable request by email to the corresponding author.

## Supplementary Materials

The following supporting information can be downloaded at: https://www.mdpi.com/article/10.3390/jcm14020525/s1, Figure S1: Funnel plots for publication bias in included studies; Figure S2: Sensitivity analysis on studies investigating pain intensity; Figure S3: Sensitivity analysis on studies investigating disability levels; Table S1: Search terms; Table S2: PEDro scores of the included studies.
