# Supplementary material for: Supervised or Home-Based? Exploring the Best Exercise Approach for Knee Osteoarthritis Management: A Systematic Review and Meta-Analysis
Source: J Clin Med. 2025 Jan 15;14(2):525. doi: 10.3390/jcm14020525 (PMC11765608; doi:10.3390/jcm14020525)
Supplement: Supplementary file 1 [file jcm-14-00525-s001.zip › jcm-3399060-supplementary.pdf]

Table S1: Search terms

Population	"knee osteoarthritis" OR "gonarthrosis"
AND	
Intervention	"home-based exercise" OR "self-management exercis*" OR "home exercise*"
AND	
Comparison	"supervised exercis*" OR "hospital exercis*" OR "supervised physical exercis*" OR "hospital-based exercis*" OR "therapeutic exercis*"
Outcomes	Pain, disability levels
Studies	Randomized controlled trials

Table S2: PEDro scores of the included studies

Studies	Random allocation	Concealed allocation	Similar at baseline	Subjects blinded	Therapists blinded	Assessors blinded	<15%dropouts	Intention-to-treat analysis	Between-group comparisons	Point measures and variability data	Scores	Quality
Alagesan et al. 2011	1	0	1	0	0	1	1	1	1	1	07/10	High
Hawkins et al., 2012	1	1	1	0	0	0	1	1	1	1	07/10	High
Tuğba et al., 2016	1	1	1	0	0	0	1	0	1	1	06/10	Moderate
Tunay et al., 2010	1	0	1	0	0	0	1	0	1	1	05/10	Moderate
Blasco et al., 2020	1	1	1	0	0	1	1	0	1	1	06/10	Moderate
Kuptniratsaikul et al.,2019	1	1	1	0	0	1	1	1	1	1	08/10	High
Kudo et al., 2013	1	0	0	0	0	0	1	0	1	1	04/10	Moderate
McCarthy et	1	0	1	0	0	1	1	1	1	1	7/10	High

al., 2004												
Jae-Young Lim et al., 2010	1	1	1	0	0	1	1	0	1	1	7/10	High
Bozgeyik et al., 2024	1	0	1	1	0	0	1	0	1	1	6/10	Moderate

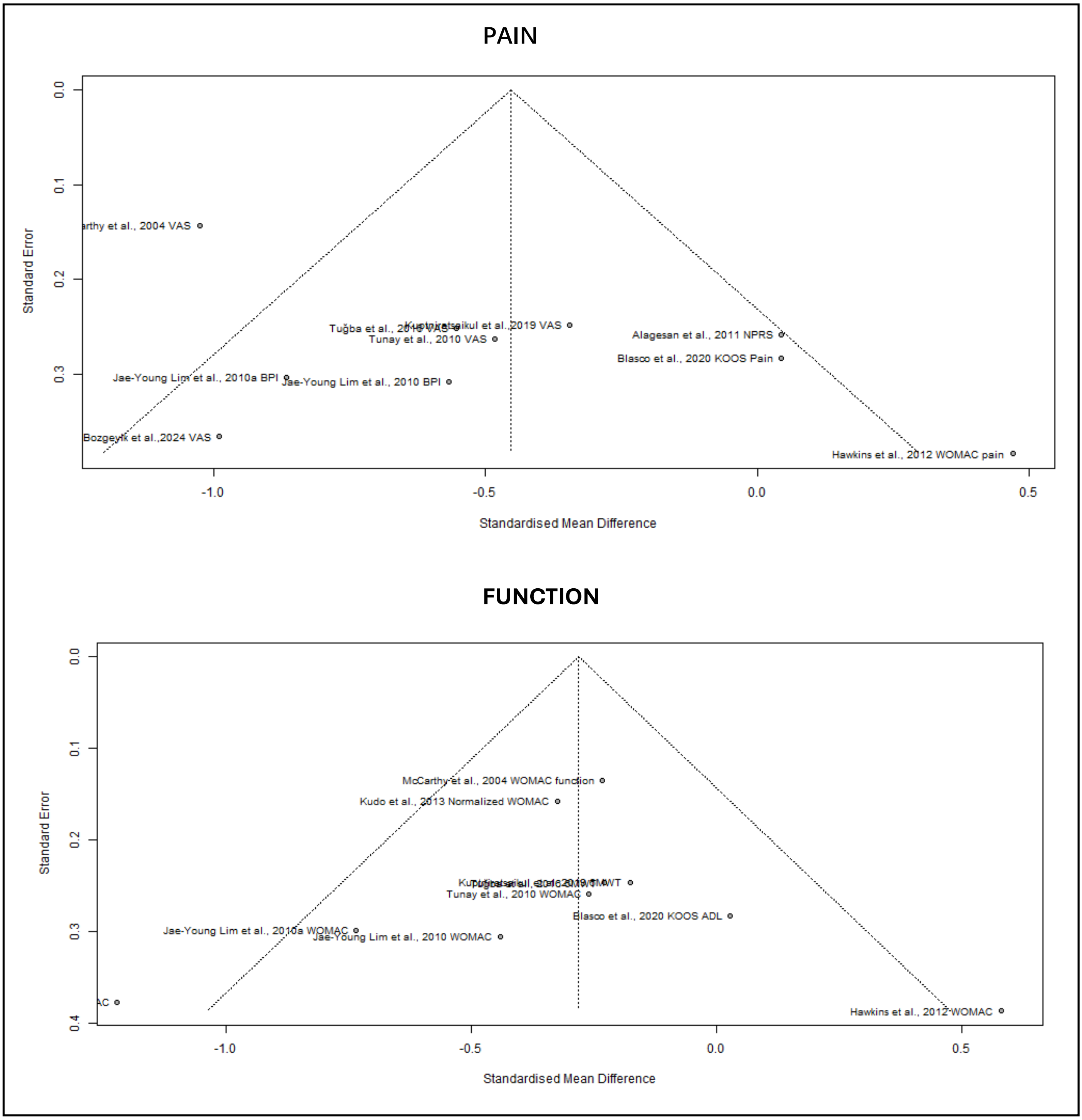

Figure S1: Funnel plots for publication bias in included studies

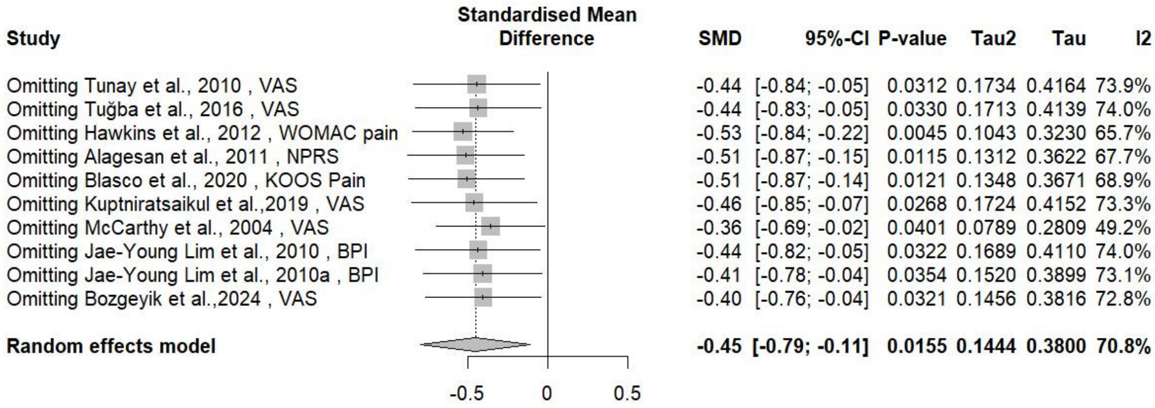

Figure S2: Sensitivity analysis on studies investigating pain intensity

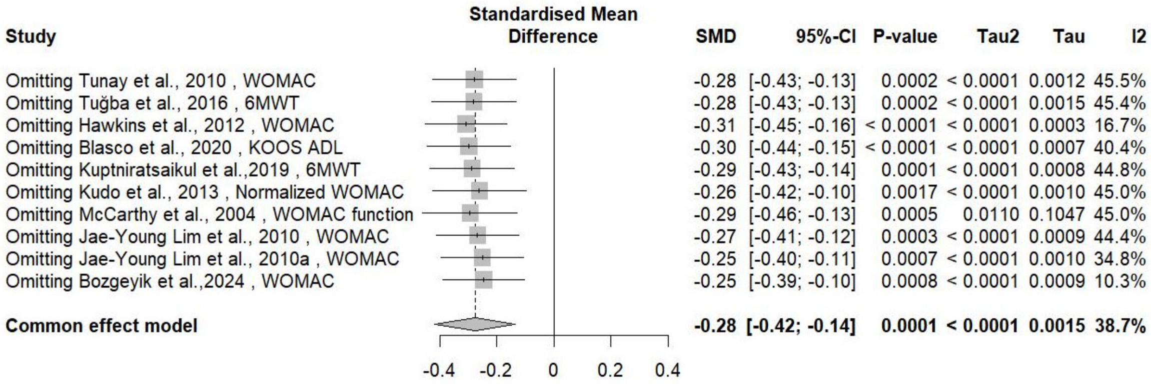

Figure S3: Sensitivity analysis on studies investigating disability levels
